# Is conduct after capture training sufficiently stressful?

**DOI:** 10.3389/fpsyg.2022.795759

**Published:** 2022-07-29

**Authors:** Niclas Wisén, Gerry Larsson, Mårten Risling, Ulf Arborelius

**Affiliations:** ^1^Department of Experimental Traumatology, Institution of Neuroscience at Karolinska Institute, Stockholm, Sweden; ^2^Department of Leadership and Command and Control, Swedish Defence University, Karlstad, Sweden

**Keywords:** SERE, conduct after capture, stress inoculation, military training, cognition, salivary cortisol

## Abstract

Conduct after capture (CAC) training is for personnel at risk of being captured. To be effective, it needs to be stressful. But how do we know if it is stressful enough? This study uses biomarkers and cognitive measures to evaluate CAC. Soldiers undergoing CAC were measured by the stress hormone cortisol from saliva samples at baseline and during training. The training consisted of being taken capture and put through a number of realistic and threatening scenarios, targeting survival strategies taught in the preceding week. Between scenarios, the trainees were held in a holding cell where they were monitored by a guard. The saliva samples were taken in conjunction with the scenarios. The whole training took place over a period of ~24 h. Cognitive performance was measured at baseline and after training. Three groups took part Group A (*n* = 20) was taken after 48 h of intense tasks leaving them in a poor resting state. Group B (*n* = 23) was well rested at CAC onset. Group C (*n* = 10) was part of a survival, evasion, resistance, and escape (SERE) instructor course. The CAC training was the same for all groups. Group A exhibited a high increase in cortisol during CAC, compared to baseline levels were multiple times as high as “expected” values. Group B exhibited elevated levels slightly lower than those of group A, they also “dropped” to “normal” levels during the latter part of the exercise. Group C displayed the least increase with only slightly elevated levels. CAC training is stressful and cortisol levels were elevated enough to satisfy the prerequisite for effective stress inoculation. No cognitive performance drop could be identified; however, several participants “froze” during the exercise due to intensive stress.

## Introduction

Stress inoculation training (SIT) is a method developed in the 1980s as a form of cognitive behavioral therapy (CBT) by Donald Meichenbaum and colleagues (Meichenbaum and Novaco, 1985). The fundamental idea of SIT is to expose a person to a stressor while providing coping strategies and methods to handle the subsequent stress successfully. The experience of successfully managing a stressful situation should “inoculate” the trainee not only against specific stressors but also other similar stressors and thus be generalizable. The idea of SIT has been transferred from the therapy environment to the military environment, where it has been used in several settings. For a comprehensive in-depth description of SIT’s modern military use, see Robson and Manacapilli (2014). SIT is also used in areas in which performance under stress is key to survival, such as Conduct After Capture (CAC). The intense stress from a life-threatening situation will activate survival responses, often labeled as fight, flight, or freeze. The survival response will mainly benefit physical performance compared to cognitive performance concerning functions such as, problem-solving, decision-making, and making use of theoretical tactical models during the response. Providing the experience of overcoming stressors and successfully making use of cognitive strategies, will according to the SIT paradigm, increase self-efficacy when faced with similar challenges.

CAC training is a form of SIT that aims to replicate the intense stress of being captured, kidnapped, or exposed to a threatening interrogation. CAC training is provided not only to military personnel but also to journalists active in unstable parts of the world as well as to ship crews traveling in waters frequented by pirates. Real-life experience, e.g., the experience of individuals who have undergone CAC training and then been taken capture, has shown at least anecdotally that the training lessens stress and increases a sense of control. As stated by a journalist taken captive in Syria in 2013, “in the middle of the stress and fear, the training provided comfort, I recognized the situations and knew what to expect” (Helmertz, 2019).

Military CAC training with the Swedish Armed Forces is a part of Survival, Evasion, Resistance, and Escape training (SERE), SERE C training. SERE A and B are basic survival training with a focus on finding food, keeping the right body temperature, and signal for help, etc., it is a part of basic soldier training. SERE C represents the highest course level, aimed at high-risk personnel. There have been several studies on CAC, which have examined stress hormones, cognitive performance, and mood among a variety of physical measures (Lieberman et al., 2016; Suurd Ralph et al., 2017). The findings include significant effects on those measures as a result of the intense stress experienced during the exercises, concluding that SERE or CAC training is stressful. However, SERE or CAC training might share the same label between nations, but the components and setting might differ in ways that calls for evaluation of the intended effect. One cannot evaluate the effect in real-life settings, e.g., being captured, other than in the unfortunate events where an individual has been taken capture. The intended effect from the training, however, is to satisfy the requisites for SIT. That is, in order to gain the self-efficacy of managing a high-stress capture situation one must pass a similar challenge with a successful outcome. Thus, our research question was as follows: Is the CAC training sufficiently stressful? If the experienced stress is too low, the requisite for SIT is not met (Suurd Ralph et al., 2017).

SERE C at the Swedish Armed Forces Survival School is a 2-week course, with the CAC event occurring within those weeks. During the exercise, trainees are exposed to different scenarios and situations that place them in stressful scenarios (interrogations) that they face alone, termed “ploys.” Between ploys, the trainees are placed in a “holding cell” (a large concrete room with no furniture), under the surveillance of a hostile guard.

CAC training has undergone continuous change over the years from a focus on physical stress (e.g., rough treatment, stress positions, and exposure to cold) to a more controlled, safer approach with more focus on psychological stress. This change warrants a structured evaluation to validate the effectiveness of the training. In this evaluation initiated by the SERE School, three consecutive groups (A, B, and C) undergoing CAC training, were examined with a focus on stress measured by cortisol and the effects of the exercise on cognition. The groups differed due to the natural selection of participants, in several ways. Two of the groups (A and B) were selected military staff that serve in high-risk position often in the air force, the third group (C) were a SERE instructor course including a full CAC training. In this study, we looked at the longitudinal data for each group firsthand. We did however compare the groups acknowledging the fact that they differed both in their demographics and the state they were in when entering the exercise. Taking that into account, we argue that the groups are similar enough to warrant a comparison on some of the identified confounders. Age is an example; Group A were younger than B and C, and most research on cortisol and ageing focus on the ageing adult (around 70 years). Research has shown that levels of cortisol are relatively stable in adulthood even though a small decline is observed during the early 20s to the 40s where it increases again (Moffat et al., 2020), making a comparison of cortisol between the groups that differ slightly in age valid. The groups also had different resting states when entering the training, sleep deprivation has been shown to affect cortisol increasing reactivity the following day (Hirotsu et al., 2015).

Since CAC is a resource-intensive training, it is important to evaluate whether the training creates the desired effects.

What is stressful enough during CAC training? Stress responses are psychological and physiological, the interaction is to some extent individual and based on previous experience and exposure to stressors as well as the appraisal of the situation (McEwen, 1998). A strong psychological stress evokes a significant physiological response including a release of stress-related hormones such as cortisol. If the training is perceived as stressful, we expect to see elevated levels that are so high that they cannot be result as over the day fluctuations to normal stress. Since a strong stress response can cause a temporary decline in cognitive performance (Lupien et al., 2007; Juster et al., 2010), instructors will adapt the stress during a ploy to a level that allows the participant to perform with sufficient function and utilize methods and strategies ending the ploy with a success. We therefore are not concerned with providing too much stress. As pointed out, changes have been made towards the exercise paradigm, that has lowered the amount of physical stressors and rough handling (due to the risk of injuries), leaving the instructor with nonphysical stressors such as shouting, isolation, false information, moral dilemmas, etc. Since psychological stressors in a training environment can be mitigated by keeping a focus on that, it is just a training exercise, it could mitigate the stress to such an extent that it loses its function as a core component in stress mitigation training.

We choose cortisol as our objective stress measure; cortisol is frequently used as a biomarker of stress because it is easy to collect from saliva and since modern technology facilitates on-site analysis. Cortisol has a relatively stable diurnal curve over time. Its peak occurs in the morning, a measurement referred to as the cortisol awakening response (CAR; Wust et al., 2000; Hellhammer et al., 2009), and then slowly declines in the course of the day (Wust et al., 2000; Matsuda et al., 2012). However, acute stress can significantly increase cortisol as a reaction to the stressor (Kirschbaum and Hellhammer, 1989; Hellhammer et al., 2009; Bozovic et al., 2013). There is, however, a rather substantial Intra Individual Variation (IIV; Segerstrom et al., 2017), meaning that the smooth slope we infer from morning to evening is not so smooth after all. Over the course of the day, there are natural fluctuations due to everyday activity and the magnitude of the fluctuation is related to the perceived magnitude of the stressor (Schlotz et al., 2011). Nevertheless, there is a clear downward slope from the morning peak to the evening–night nadir. In addition to psychological events, variations in rest, food intake, nicotine, coffee, and other substances can affect daytime cortisol levels (Kudielka et al., 2009). There are limitations to using cortisol to measure minor effects of stress due to IIV. Previous studies (Morgan et al., 2000; Suurd Ralph et al., 2017) show that the effects of CAC go well beyond what could be expected from IIV.

SIT builds on successfully using learned strategies in a stressful situation where access to and execution of the strategies is perceived as hard or demanding due to loss of ability to use one’s full cognitive potential. Therefore, it is relevant to assess how cognition is affected by the CAC training. Here, cognition is defined as a mental action of processing information in the brain with the goal of producing a favorable response. It is impacted by stress in several ways. It affects recall (memory) and problem solving as well as perception. Stress impacts cognition following the Yerkes–Dodson law (Yerkes and Dodson, 1908), that is that an optimal level of stress will increase performance, while too little or too much stress will result in less than optimal performance. Since it follows an inverted u-shape, it implies that we need some stress to recruit resources in order to perform at peak level. However if stress is too intense, we pass the peak and our performance declines with increased stress or load. Measuring cognitive performance can be done using Reaction Time measures which had been previously utilized in similar studies (Harris et al., 2005).

## Question/Hypothesis

Based on the literature and the course setup, we designed an evaluation study with the following hypotheses:

A/ CAC training will increase psychological stress, as measured by salivary cortisol, during the exercise compared to baseline measures.

B/ CAC training will have a negative impact on cognitive performance, assessed directly after the exercise, compared to a baseline assessment.

C/ Explorative: Will there be a difference between the groups.

## Materials and methods

### Design

Data were collected as an evaluation of the CAC part of the SERE C course. Using the design described below, the collected data were subsequently included in this study for scientific evaluation. There are several ploys during the CAC event. We chose to sample saliva from four evenly spaced ploys over the entire period of captivity which provided us a spread of data over time that were comparable to the baseline measures and normal fluctuations of cortisol levels during a 24 h cycle. Salivary cortisol was collected right after the ploys. The ploys had the same setup for all the trainees. However, the ploys unfold partly due to the interaction of the trainee. Therefore, they differ in intensity and length. The nature of the exercise, which is supposed to represent a relatively novel and unknown situation for the participants, prevents us from describing the setup in detail. It is a 2-week course; in the first week, they are taught methods and strategies to increase the likelihood of survival. How to create value and how to use information strategically to keep you an asset over time. During the CAC exposure exercise, its put to a test how effectively the participants can apply the methods and strategies taught the previous week. The exposure training covers 24h starting with participants taken capture, they are then put through the different plojs and supervised confinement both together with peers and in solitary confinement. The aim of the CAC part of the course is to put participants through a challenging setup where the theoretical foundation taught at week one is put into a practical test. As described unless a participant fails completely, they will be guided thorough the ploys in a way that lets them experience that they can perform under pressure.

### Participants

The number of participants was as follows: Group A (*n* = 20), group B (*n* = 23), and group C (*n* = 10). In total, there were 53 participants in the study, (all participants of the course) and there was one female participant. The age distribution (years) for the groups was as follows: A (mean *=* 24.4, SD = 7.1), B (mean = 28.8, SD = 3.91), and C (mean = 28.9, SD = 4.2).

The participants were from all branches of the armed forces: Air Force (*n =* 45), Army (*n =* 7), and Navy (*n* = 1). The participants in groups A and B underwent the training as part of their ordinary training (mandatory), while group C participants had applied for the longer SERE instructor course (voluntary). This study was initiated as a training evaluation study and participation was expected still they were verbally informed on that they were tested. Subsequent use of these data for a scientific approach has been subject to ethical evaluation by the Swedish Ethics Review Authority, (review nr: 2019-05361). The committee concluded the data do not meet the requirements to be subject for individual consent to be used, since no identification from data is possible.

### Grouping

Trainees from three SERE C courses (groups A, B, and C) were all included in the CAC evaluation. Group A entered the CAC after a period of 48 h containing an “urban evasion” training where they are to avoid capture by instructors in a small Swedish town. The night before the CAC exercise they also were put through a heat chamber exercise covering several hours. They were taken capture when going back to have the next day off resulting in limited sleep and food intake. Group B was given the night before exercise off, ending at around 6 p.m. They were well rested and fed when entering the CAC exercise the following morning. Group C differed from the others in that its members were trainees undergoing an instructor course for the SERE A and B levels. The instructor course is longer than the SERE C course. Thus, the participants have an opportunity to get to know one another. Compared to the other groups, group C consisted of a wider array of individuals from the different branches of the armed forces (five Army, one Navy, four Air Force) than group A (two Army, 18 Air Force) and group B (23 Air Force). The previous experience with SERE, however, did not include CAC training. Group C also entered the CAC exercise well rested and fed. Table 1. Group specifics.

**Table 1:** Group specifics.

Group	*n*	Age mean	Branch	Specifics
A	20	24.4, SD = 7.1	Two Army 18 Air Force	Pre exhausted (sleep and food deprived) the 24 h before preceding capture.
B	23	28.8, SD = 4.0	23 Air Force	Well rested before capture
C	10	28.9, SD = 4.2	Five Army One Navy Four Air Force	Well rested before capture. Voluntary participation in course, and previous similar experience.
tot	53	27.1, SD = 5.8	One Navy Seven Army 45 Air Force	

### Procedure

The three groups A, B, and C were all subjected to baseline testing the week before the CAC exposure. The baseline tests were given on a “lecture” day starting at wake up collecting three saliva samples (at awakening and after 15 and 30 min) to obtain the CAR, and then at ~06:00 p.m. to measure the evening level. Drinking, food intake, nicotine use and teethbrushing were prohibited during the 30 min prior to saliva collection to avoid their affecting the saliva content. During CAC, saliva was collected after each of the 4 ploys selected for sampling. Since all participants were subjected to the same ploys in a consecutive manner, the sampling time varied within the range of each ploy (i.e., 1–2 h) and depending on participant performance. The saliva collected from the ploys are referred to as sample events 3–6. The Ploys were evenly distributed during the day staring at around 9 a.m. (after the capture and incarceration procedure). Last ploy were sampled around 8–10 p.m.

Two cognitive tests were given, one at the same lecture day as the cortisol sampling. Follow-up testing was performed right after the end of the exercise.

### Variables

#### Stress

The main stressor that is applied through the training is the psychological stress provided by the CAC exercise. Since physical stressors are excluded (no stress positions, rough handling, cold or heat exposure, etc.,) all remaining stressors are psychological in nature. The training environment is designed to be realistic, that is in isolation, filled with uncomfortable smell and sounds, participants are also put into captive clothing’s and from time to time they wear a hood covering their eyes. The handling from instructors is mainly shouting, threatening, degrading, or trying to play participants against one and other and to take away the feeling of being in control of the situation. There is a component of pass or fail that can be a source of stress; the participants are not aware that the instructors will adapt and guide them through the ploys since the idea is not to test the individuals but to have them experience that they made it even though it took some effort.

#### Initial “rest” status

The second impact factor was the initial resting state of the group when entering the CAC exercise. Group A were in a status of sleep and food deprivation. While Group B and C were well rested and feed. The factor was not measured due to the training schedule. Group A were doing overnight training the preceding 48 h, while group B and C finished at noon the day before the CAC training.

### Data collection procedures

#### Cognitive measurements

The participants were given a digital cognitive test based on reaction time (RT) measurements. They were given the test at twice once at baseline and once directly after the exercise. The cognitive test battery consisted of three RT-based cognitive tests as follows. Simple reaction time (*SRT*) is defined as the time required to elicit a simple defined reaction to a stimulus, often using a visual stimulus with a motoric response. SRT is assessed by touching a dot (stimuli) as fast as possible on a screen when the stimulus appears, the test covers 20 stimuli events (dots) with randomly varied intervals between. Choice reaction time (*CRT*) adds stimuli identification and response selection to the SRT paradigm, compared to the SRT, the test has four independent symbols. When they appear on the screen, the respondent touches the “button” with the corresponding symbol on it. The response buttons have two symbols each and are situated below the area where the stimuli appear. As with SRT, the test has 20 events with varied time intervals between. The go or no go (*GNG*) test paradigms present two different stimuli appearing in a grid with six possible places for the stimuli to appear. When a blue dot appears in one position in the grid the correct response is to refrain (inhibit) a response, while a red dot is correctly responded to by touching the “shoot” button, the test covers 10 response stimuli and 10 inhibit stimuli randomly distributed over the test (Littman and Takács, 2017).

RT-test paradigms are a well-established way to measure information-processing performance (Woods et al., 2015; Burke et al., 2017), and SRT has been shown to be a valuable test paradigm in measuring stress-induced deterioration (Harris et al., 2005). Our version was given on an Android-based tablet, using a program developed inhouse based on well-established test protocols and paradigms, for bringing test availability to the field.

#### Biological measurements

Cortisol was used as a biomarker for stress. The saliva was sampled using Salivette™ collectors. Cortisol analysis was performed using mobile salivary cortisol assays (I-calQ, LLC; Scottsdale, Arizona, United States). The I-calQ is developed for field use (medical), which makes it possible to test the collected saliva onsite with no storage or delays still they were kept frigerated during the exercise before analyzed.

It uses the immunoassay test strips and image analysis algorithm to analyze the saliva. The cortisol assay utilizes affinity chromatography. That is Antibodies developed with a high affinity for particles of cortisol, These antibodies adhered to cortisol produce a visible signal. The intensity of this signal correlates with the amount of cortisol present in the saliva sample, which is also correlated to the blood concentration of cortisol.

### Statistics

Cognitive measures were analyzed using a MANOVA repeated-measure design covering between-group and within-group baseline-post-measurements, comparisons. The use of a MANOVA was motivated by the assumption that all cognitive subtests measure an underlying function that could indicate an overall effect.

Cortisol measures were compared based on group means and complemented with analysis of AUC area under the curve.

Statistics were analyzed using, SPSS version 26 and R.

## Results

### Cognitive measures

Table 2 presents means and standard deviations of the three groups on both measurement occasions. The Shapiro–Wilk statistic shows that the response distributions of all three reaction time tests were statistically non-normal on the first assessment. On the second measurement occasion, the three tests did not deviate significantly from normal. This was confirmed by the Kurtosis values. On the first measurement occasion, they ranged from 1.034 to 1.404. On the second assessment, they ranged between 0.511 and 0.594.

**Table 2:** Between-group comparison one-way analysis of variance.

								
Variable	Group A (*n* = 20)		Group B (*n* = 23)		Group C (*n* = 10)		Variable Shapiro–Wilk	
	*M*	SD	*M*	SD	*M*	SD	Statistic	*p*
**TIME 1**								
Simple reaction time^a^	335.25	31.02	359.29	46.46	349.59	25.41	0.904	0.001
Choice reaction time^a^	707.30	87.96	773.49	129.61	724.31	95.21	0.919	0.002
Go or no go^a^	461.40	54.00	514.21	69.46	487.22	46.38	0.941	0.014
**TIME 2**								
Simple reaction time^a^	350.40	46.95	360.36	38.55	349.55	35.36	0.959	0.079
Choice reaction time^a^	753.95	143.44	759.80	89.48	740.15	65.92	0.963	0.115
Go or no go^a^	472.85	53.90	517.33	44.40	498.78	74.54	0.961	0.096

a Scores show ms.

**Figure 1:**
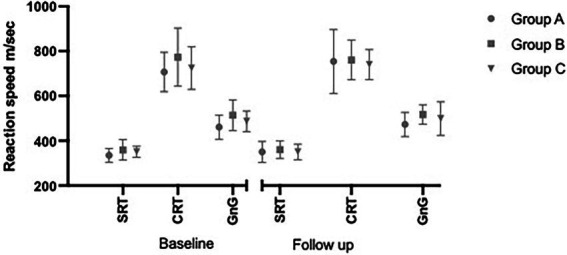
Cognitive test for all groups A–C at baseline and follow-up; the vertical bars represent one standard deviation. SRT, simple reaction time; CRT, choice reaction time; GnG, go or no go.

A MANOVA repeated-measures design was used to test within-and between-group differences on the cognitive tests. Since there were only two levels (time and group), the assumption of sphericity was met and the Mauchly’s test was not applicable (Field, 2000). The Box’s *M*-test score was 62.20, *F* = 1.156, (42, 2839,38), *p* = 0.228, indicating that the observed covariance matrices of the three reaction time tests were equal across the three groups. Mahalanobis’ distance showed one extreme value which caused the critical value for six dependent variables (the three cognitive tests pre-and post) to slightly exceed the maximum limit (22.72 where the limit is 22.46). Beginning with within-subjects effects across time, the multivariate tests (Pillai’s trace) did not show any significant differences. Turning to the between-subjects effects, also here no significant differences emerged the graphical distrubution is shown in Figure 1.

### Biological measures

**Figure 2:**
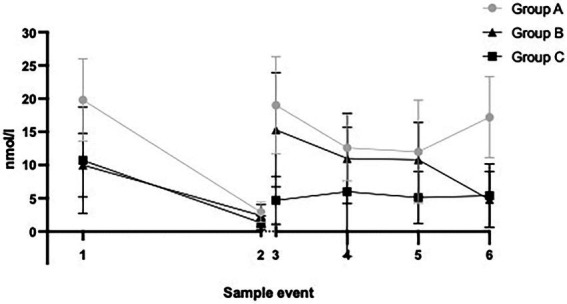
All groups over all events: 1–2 baseline, 3–6 during CAC (vertical bars one SD).

The cortisol values are presented in Figure 2 using the mean nmol/l. The sample events refer to the time the samples were collected. Sample event 1 is Baseline CAC (the mean of the three awakening measures), and Sample event 2 is the baseline pm measure (covering ~12 h during the day). Sample events 3–6 are the measures taken after each ploy during CAC. The diurnal fluctuation of cortisol with its peak in the morning and the nadir in the evening gives an estimate of the slope over the day. The graph in the figure shows that the baseline levels follow that natural decrease during the day. Sample events 3–6 show a different path, with levels being elevated throughout the exercise. Cortisol levels were also compared using the area under the curve (AUC), it also shows a significant elevation during the CAC training. AUC was calculated for individuals with a full dataset (all measures), 8 individuals had non-complete tests due to not sufficient saliva when sampling. Therefore, AUC results are based on 45 individuals. The mean AUC for each group at baseline, training, and the absolute difference is presented in Table 3. An overall repeated-measures ANOVA showed significant differences between groups, time, and group:time. Pairwise comparisons over time (within each group) show that groups A and B have a significant difference in AUC, while group C does not Tables 4, 5.

**Table 3:** Area under the curve (AUC) for all groups A, B and C at baseline and during Conduct after training (CAC).

Group	Variable	*n*	Mean	SD
A	AUC at baseline	19	34.04	10.43
B	AUC at baseline	21	18.59	7.42
C	AUC at baseline	10	17.94	12.96
A	AUC during CAC	19	42.73	13.03
B	AUC during CAC	23	30.43	13.32
C	AUC during CAC	10	15.67	9.70
A	Absolute difference in AUC	19	8.69	13.00
B	Absolute difference in AUC	20	13.36	13.78
C	Absolute difference in AUC	10	−2.27	13.06

The last three lines show the difference between the two times.

**Table 4:** Overall repeated measures ANOVA.

Effect	*dfn*	*dfd*	*F*	*p*
group	2	46	22.616	0.000
time	1	46	10.848	0.002
group:time	2	46	4.587	0.015

**Table 5:** Pairwise comparisons between time points (within each group).

omg	Time 1	Time 2	n1	n2	Statistic	*df*	*p*
A	Baseline	Post	19	19	−2.91	18	0.0090
B	Baseline	Post	20	20	−4.34	19	0.0004
C	Baseline	Post	10	10	0.55	9	0.5960

## Discussion

The results support the main hypothesis that cortisol levels increase during the exercise with the exception for group C. The cortisol taken at baseline affords an estimate of a normal decline of levels during a day without intense stress (Cochrane et al., 2014). Based on the baseline measures, we can compare the assumed “slope” with the values from the exercise, as presented in the graphs. The soldiers in all groups exhibited increased levels over the entire period of captivity. This phenomenon might not be as obvious in the morning when the system is naturally saturated. However, comparing the evening (baseline late sample) and the third test event (taken during the afternoon/evening ploy), we found elevated levels during CAC 4–5 to be times higher than baseline levels. The results compared between groups both using means and AUC indicate that there were effects on cortisol levels from preceding stress, such as sleep deprivation and low food intake. Group C however did not show the same profile, they were more prepared, had a more established team feeling, and were voluntary participants, all factors that could possibly mitigate the stress response during CAC. However, the first measure (CAR) was so low that one can suspect an possible artifact in sampling due to some error in reading, testing equipment, or other factors that influenced all the samples taken at that time.

The cognitive tests performed before and after the exercise indicated no change in cognitive processing speed. Although the response distributions on the three reaction time tests deviated from normality on the first test occasion, the deviance was limited and they met the normality requirement on the second assessment. Thus, we regard the use of the MANOVA repeated-measures design as legitimate. RT tests are commonly used to measure cognitive performance. However, the ability measured requires a significant stress to show any decline in performance. This is a challange for military perfromance research in general. Its hard to find test that has ecological validity and that test clos to what would be an actual responce in a real-life situation. During the exercise, the participants were observed to have difficulties accessing the methods and strategies they were taught the preceding week. At an extreme, one soldier became “stuck” for over 45 min in a ploy that usually required <10 min to pass. He struggled to handle the stress and find the correct responses, even with instructors providing “hints” in their role-play. One plausible explanation is that the observed decrease in cognitive performance only occurs while the participant is exposed to the stressor, and the recovery time for cognitive function is immediate. This outcome calls for improved understanding and future testing. Cognitive function is a broad area, and when it appears to fail in a situation that makes use of taught models (i.e., recall and activation), we must determine which properties are responsible for the “freeze” or lack of access to cognitive resources. The ambition to bring participants through CAC training with a feeling of success is the reason for instructors “hinting” or leading them through the ploys, one could argue that it might affect the stress response and following cortisol sample. The “hinting” or “support” is however not obvious and the instructors are trained at keeping their hostile approach even when offering a way out by presenting obvious use of the tools they have been taught.

Since we did not have performance measures for each ploy, we cannot compare ploy success with cortisol levels. Could individuals with higher cortisol response be more prone to perform worse than those who had a lower response, an indication of less perceived stress? This question can be addressed in future research. Further, there are other limitations to this study, since it was performed on already planned groups and curriculums the only thing we could affect was the resting state at onset. Therefore, there is no random assignment between groups, and Group C differs in many aspects of the group composition.

## Conclusion

We hypothesized that the CAC exercise would increase stress to such an extent that it could be measured in salivary cortisol, there would be a cognitive performance drop directly after the exercise, and the magnitude of these effects would be affected by the rest and food status of the participants at the time of exercise onset. The results supported the cortisol hypothesis but not the cognitive performance hypothesis. Saliva was easy to collect with little impact on the exercise. However, cognitive ability in the form of RT tests cannot be used during the exercise without interrupting and exerting a negative effect on exercise realism. What we observed was that the soldiers who were sleep- and food-deprived had the highest levels of cortisol reaction, indicating a higher stress response. Therefore, pre-exhaustion of participants might be a way to amplify the intended stress effect on participants with less intense stress stimuli. There is, however, a risk of less learning when sleep deprived (Pierard et al., 2004). As noted, the fundamental idea of SIT and CAC is to create a stress exposure in response to which learned skillsets can be successfully applied. Instructor reports and ploy observations revealed that there are temporary cognitive limitations due to stress. Ploys are fairly standardized, and it should be possible to find ways to assess cognitive performance during each ploy. Such a design could possibly identify which cognitive components are most affected by stress. And if there are individuals who are more resilient or susceptible to CAC stressors. This question warrants further research and could be helpful in the further development of CAC training. Studies such as this one are relevant in that we must evaluate and validate training paradigms to develop them further. Operational demands and training regulations might have an impact on their intended effects (regulations and limitations). Therefore, because such training comes at a great cost for the organization providing it and for the participant, constant training evaluation is required.

## Data availability statement

The data that support the findings of this study are openly available in Zendo.org at: https://doi.org/10.5281/zenodo.4543557.

## Ethics statement

Ethical review and approval were not required for this study in accordance with the national legislation and the institutional requirements. Written informed consent for participation was not required for this study in accordance with the national legislation and the institutional requirements.

## Author contributions

NW conducted the study, analyzed the results, and wrote the paper. MR and UA provided the resources and facilitated the study. GL supervised and helped with analysis aside from providing continuous feedback thought the writing process. All authors contributed to the article and approved the submitted version.

## Conflict of interest

The authors declare that the research was conducted in the absence of any commercial or financial relationships that could be construed as a potential conflict of interest.

## Publisher’s note

All claims expressed in this article are solely those of the authors and do not necessarily represent those of their affiliated organizations, or those of the publisher, the editors and the reviewers. Any product that may be evaluated in this article, or claim that may be made by its manufacturer, is not guaranteed or endorsed by the publisher.
